# A new method for predicting the microvascular invasion status of hepatocellular carcinoma through neural network analysis

**DOI:** 10.1186/s12893-023-01967-y

**Published:** 2023-04-28

**Authors:** Jinli Zheng, Xiaozhen Wei, Ning Wang, Xingyu Pu, Jiayin Yang, Li Jiang

**Affiliations:** 1 Liver Transplant Center, Transplant Center, West China Hospital of Sichuan University, Chengdu, Sichuan Province China; 2 Department of Liver Surgery, General Surgery, West China Hospital of Sichuan University, Chengdu, Sichuan Province China; 3 Department of Hepatobiliary Department, West China Jintang Hospital Sichuan University, Chengdu, Sichuan China; 4 Department of Anesthesia & Operation Center, West China Hospital of Sichuan University, Chengdu, Sichuan China

**Keywords:** Hepatocellular carcinoma, Microvascular invasion, Neural network analysis

## Abstract

**Aims:**

To determine the relationship between microvascular invasion (MVI) and the clinical features of hepatocellular carcinoma (HCC) and provide a method to evaluate MVI status by neutral network analysis.

**Methods:**

The patients were divided into two groups (MVI-positive group and MVI-negative group). Univariate analysis and multivariate logistic regression analysis were carried out to identify the independent risk factors for MVI positivity. Neural network analysis was used to analyze the different importance of the risk factors in MVI prediction.

**Results:**

We enrolled 1697 patients in this study. We found that the independent prognostic factors were age, NEU, multiple tumors, AFP level and tumor diameter. By neural network analysis, we proposed that the level of AFP was the most important risk factor for HCC in predicting MVI status (the AUC was 0.704). However, age was the most important risk factor for early-stage HCC with a single tumor (the AUC was 0.605).

**Conclusion:**

Through the neutral network analysis, we could conclude that the level of AFP is the most important risk factor for MVI-positive patients and the age is the most important risk factor for early-stage HCC with a single tumor.

## Introduction

Hepatocellular carcinoma (HCC) ranks third in cancer-related death worldwide, with 22 new patients per 100,000 and approximately 21 deaths per 100,000 in China [1, 2]. Treatments for HCC mainly include liver transplantation (LT), hepatic resection (HR), radiofrequency ablation (RFA), and transarterial chemoembolization (TACE). Although LT is the optimal treatment for patients with HCC in the early stage [3, 4], the shortage of organs limits its feasibility [5]. Thus, hepatectomy is still the first-line treatment for resectable HCC [6]. On the other hand, the overall survival (OS) rate is still unsatisfactory due to the high rate of tumor recurrence after hepatectomy, exceeding 70% at 5-year cumulative recurrence rates [7–9]. Previous studies have reported that microvascular invasion (MVI) is an important risk factor for tumor recurrence [10–13]. Thus, an increasing number of studies have focused on searching different methods to predict MVI status. Some studies have reported that when the tumor diameter is larger than 4 cm, the tumor is likely MVI positive [14, 15]. Furthermore, a survey carried out by Yan-Yan Wang et al. showed that a tumor diameter greater than 7 cm could be more accurate in predicting the prognosis of HCC patients [16]. Thus, the relationship between the diameter of the tumor and MVI status needs further research.

Certainly, with the development of techniques and radiology skills, radiologists can predict MVI status based on radiomics [17–19]. However, there are several limits for these studies in the clinic. Based on the regular or irregular margin of the tumors to identify the MVI status, an experienced radiologist is needed and is likely affected by self-awareness [17, 18]. The efficacy of predicting MVI status is excellent; however, the tender steps and complex operations are obstructions for these methods in the clinic [19]. A previous study did not analyze the risk factors for MVI in early-stage HCC because several studies reported that the long-term outcomes of RAF were comparable to those of hepatectomy for early-stage HCC [20, 21]. Thus, we performed a retrospective study to detect an effective method to conveniently evaluate MVI status and provide an approach for surgeons to make decisions on HCC, especially for early-stage HCC.

## Patients and methods

### Patients

The study retrospectively enrolled patients with HCC in our center, Department of Liver Surgery and Liver Transplantation Center, West China Hospital of Sichuan University, from January 2012 to January 2020. The patients were enrolled as follows: (1) age > 18 years old, including males and females; (2) no other tumor therapy history, especially the recurrence of HCC; (3) no other fatal disease, such as heart disease and respiratory insufficiency; (4) no LT treatment; and (5) Child‒Pugh class within A and B. If the patient could not meet one of the following criteria, he or she was excluded.

### Methods

The patients were divided into two groups (MVI-positive group and MVI-negative group). Univariate and multivariate logistic regression analyses were carried out to identify the independent risk factors for MVI positivity, and we took the variables into multivariate logistic regression analyses when the p value was less than 0.2 [22]. Finally, we performed neural network analysis for independent risk factors to analyze the different importance of the risk factors in MVI prediction.

The patients with HCC and MVI positivity were diagnosed by a histopathological examination after hepatic resection. MVI was defined as a tumor within a vascular space lined by endothelium that was visible only via microscopy, including hepatic vein, hepatic artery and bile duct invasion [23, 24].

### Preoperative evaluation

All patients were informed about the treatments, including LT, HR, RFA and TACE. Liver function, blood tests, coagulation function and imaging examinations were reviewed by surgeons with more than 5 years of experience in hepatectomy.

### Statistical analysis

SPSS 22.0 statistical software (SPSS Inc., Chicago, IL, USA) was used to analyze the relevant data. The categorical data were presented as the number (percent) and compared using Pearson chi-square or Fisher’s exact test. The continuous variables were expressed as the mean value ± SD and were analyzed by the T or W test. Based on the outcomes of the T test, Pearson chi-square test or Fisher’s exact test, we performed a multivariate logistic regression analysis to test potential predictors of MVI status. A 2-tailed P < 0.05 was considered statistically significant. Neural network analysis was used to clarify the effect of the independent risk factors on MVI status. The neural network analysis exited randomness, which could not be avoided because the initial parameters were random at the beginning every time, meaning we could obtain thousands of prediction models. Certainly, we performed the neural network analysis more than 100 times and selected one of the results in the maximal area under the curve (AUC). We also performed receiver operating characteristic (ROC) curve analysis to compare the efficacy of these independent risk factors.

## Results

### The characteristics of the patients

This study enrolled 1697 patients with HCC undergoing HR, including 235 MVI-positive patients and 1462 MVI-negative patients. Among these patients, 1352 were male and 345 were female. Table 1 shows the characteristics of the patients in these two groups. The significant differences between these groups were age (52.09 ± 13.43 versus 49.15 ± 11.99, *p* = 0.002), NEU (59.08 ± 18.66 versus 63.30 ± 13.62, *p* = 0.001), serum AFP (*p* < 0.001), tumor numbers (*p* < 0.001) and tumor diameters (5.39 ± 3.14 versus 7.01 ± 3.01, *p* < 0.001). There were no deaths within 30 days after the operation.

**Table 1:** The characteristic features of patients

	MVI negative (N = 1462)	MVI positive (N = 235)	*p*
Sex (male, ratio)	1152 (78.80%)	200 (85.11%)	0.026
Age (years)	52.09 ± 13.43	49.15 ± 11.99	0.002
HGB (g/L)	139.16 ± 22.30	140.53 ± 21.68	0.210
WBC (× 10^9^ /L)	6.48 ± 3.01	6.20 ± 2.91	0.742
PLT (× 10^9^ /L)	139.65 ± 78.25	139.78 ± 76.10	0.981
NEU (%)	59.08 ± 18.66	63.30 ± 13.62	0.001
CHB (positive, ratio)	1102 (75.38%)	186 (79.15%)	0.209
Serum AFP (≥ 400 ng/mL, ratio)	500 (34.20%)	135 (57.45%)	< 0.001
TB (µmol/L)	18.88 ± 6.10	17.86 ± 5.06	0.588
AST (median IU/L)	45	47	0.231
ALT (median IU/L)	37	39	0.373
ALB (g/L)	41.07 ± 6.23	40.53 ± 5.40	0.215
PT (s)	12.00 ± 1.35	11.94 ± 1.45	0.588
Tumor type (single, ratio)	1261(86.25%)	184(78.30%)	0.001
Diameter (maximal tumor)	5.39 ± 3.14	7.01 ± 3.01	< 0.001

HGB:Hemoglobin; WBC: Withe Blood Cell; PLT:Platelet; NEU: Percentage of Neutrophils; CHB: Chronic Hepatic B Virus; AFP:ɑ-fetoprotein; TB: Total Bilirubin; AST:Aspartate aminotransferase; ALT:Alanine aminotransferase; ALB: Albumin; PT: Prothrombin Time

### Independent risk factors for patients

**Fig. 1:**
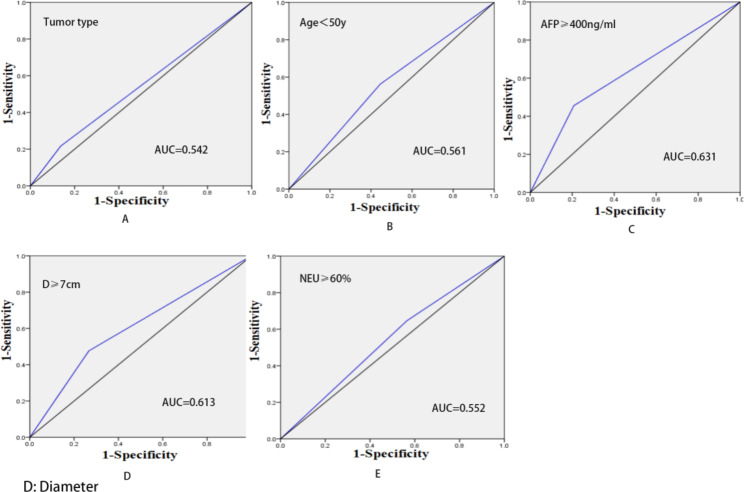
The ROC of independent risk factors in predicting MVI positivity

According to Table 1, we performed a multivariate analysis (Table 2) and found that the independent prognostic factors were age, NEU, multiple tumors, AFP level and tumor diameter. Among these independent risk factors, we found that age was a protective prognostic factor for these patients, meaning that elderly patients are likely MVI negative, and the level of serum AFP was the most important risk factor for MVI positive (Table 2). We also performed ROC curve analysis for these independent risk factors (Fig. 1) and found that the level of AFP was more effective in predicting MVI than other risk factors. (Fig. 1-C, with ACU = 0.631)

**Table 2:** Univariable and multivariable analysis of the prognostic factors for MVI

	Univariable				Multivariable		
	OR	95%CI	*p*		OR	95%CI	*p*
All patients							
Age (≥ 50 years)	0.525	0.353–0.834	0.001		0.527	0.471–0.807	0.001
Sex (Male)	1.238	1.008–2.149	0.037				
PLT (≤ 100 × 10^9^/L)	1.41	0.825–1.554	0.149				
Maximal tumor size (≥ 7 cm)	2.203	1.910–3.215	< 0.001		2.005	1.170–2.521	< 0.001
AFP (≥ 400 ng/mL)	3.098	2.302–4.057	< 0.001		2.513	2.193–3.582	< 0.001
Tumor (multiple)	1.539	1.333–2.253	0.005		1.758	1.099–2.258	0.001
NEU (≥ 60%)	1.210	1.009–1.778	0.012		1.252	1.001–1.608	0.024
Patients in early-stage with single tumor							
Age (≥ 50 years)	0.550	0.396–0.752	0.031		0.678	0.396–0.885	0.036
Sex (Male)	5.479	0.766–8.492	0.099				
PLT (≤ 100 × 10^9^/L)	1.045	0.618–2.431	0.638				
AFP (≥ 400 ng/mL)	1.702	1.106–2.164	0.001		1.342	1.110–3.700	0.024
NEU (≥ 60%)	1.371	1.124–1.954	0.046		1.301	1.199–2.812	0.045

### Neural network analysis for independent risk factors

**Fig. 2:**
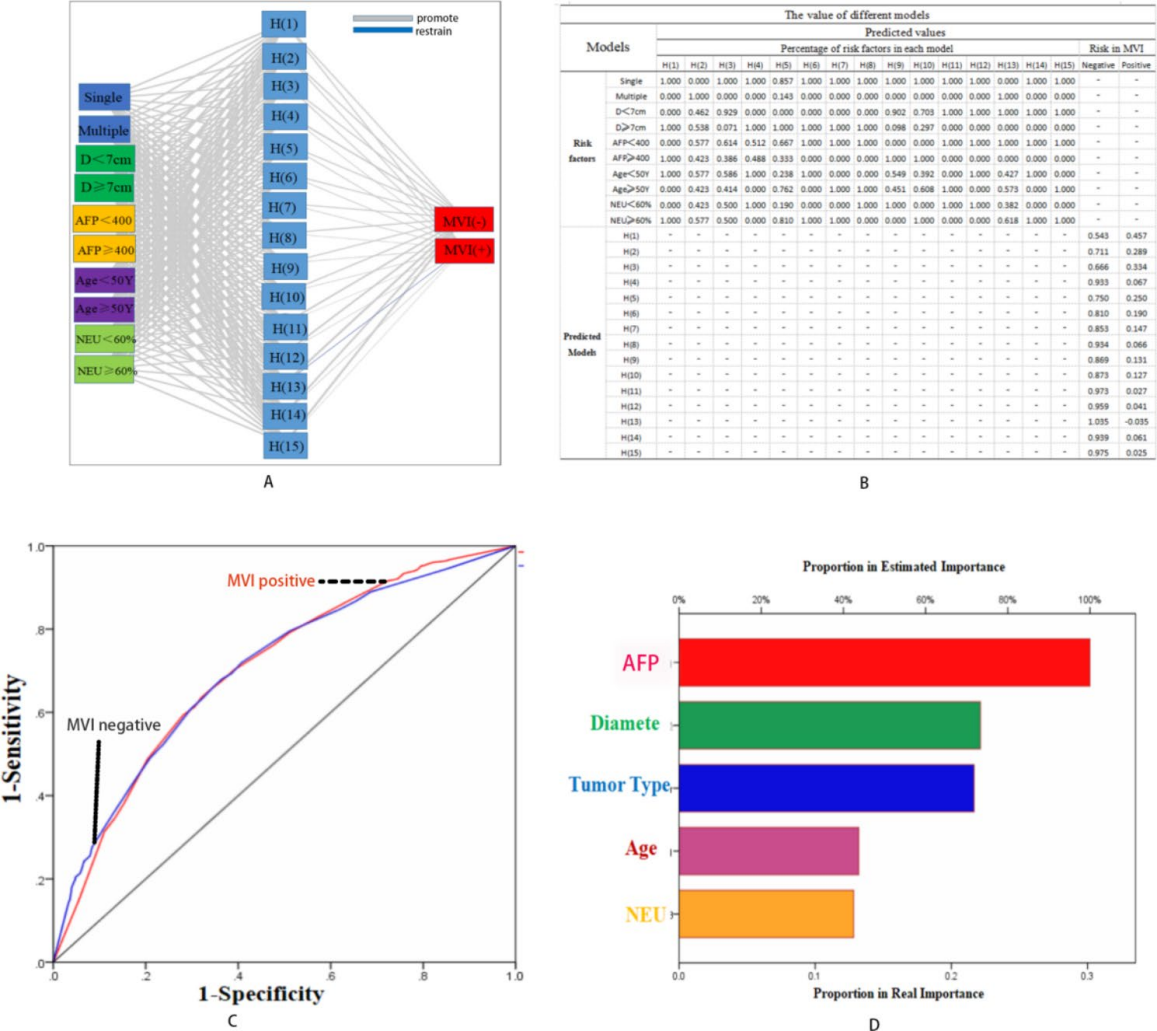
Neural network analysis for independent risk factors in these patients

The neural network analysis was performed by independent risk factors, and the results showed that AFP was the most important risk factor for predicting MVI status (Fig. 2-D). The AUC was 0.704 (Fig. 2-C). Figure 2-A shows that the independent risk factors have different effects in predicting MVI status. As shown in Fig. 2-B, the neural network analysis built 15 models for predicting MVI status. Combined with Fig. 2-B, we could explain these models simply. When a patient had a single tumor, a tumor diameter less than 7 cm and a serum AFP level less than 400 IU/L, he was more likely to be MVI negative (H (11), H (12), H (14) and H (15)). On the other hand, when a patient had a single tumor and tumor diameter larger than 7 cm, if the serum AFP was higher than 400 ng/mL, he would be more likely to be MVI positive than if the serum AFP was less than 400 ng/mL (H(1) compared to H(6), H(7) and H(8)). For multiple tumors, when the diameter of the largest tumor was less than 7 cm and the serum AFP level was less than 400 ng/mL, the patient tended to be MVI negative (H (13)). On the other hand, the risk factors for age and NEU were weakly related to MVI status, which was coincident with the multivariate analysis (Table 2). Thus, we could roughly predict the MVI status through the diameter of the tumor, the level of serum AFP and the number of tumors.

### Neural network analysis in early-stage single tumors

**Fig. 3:**
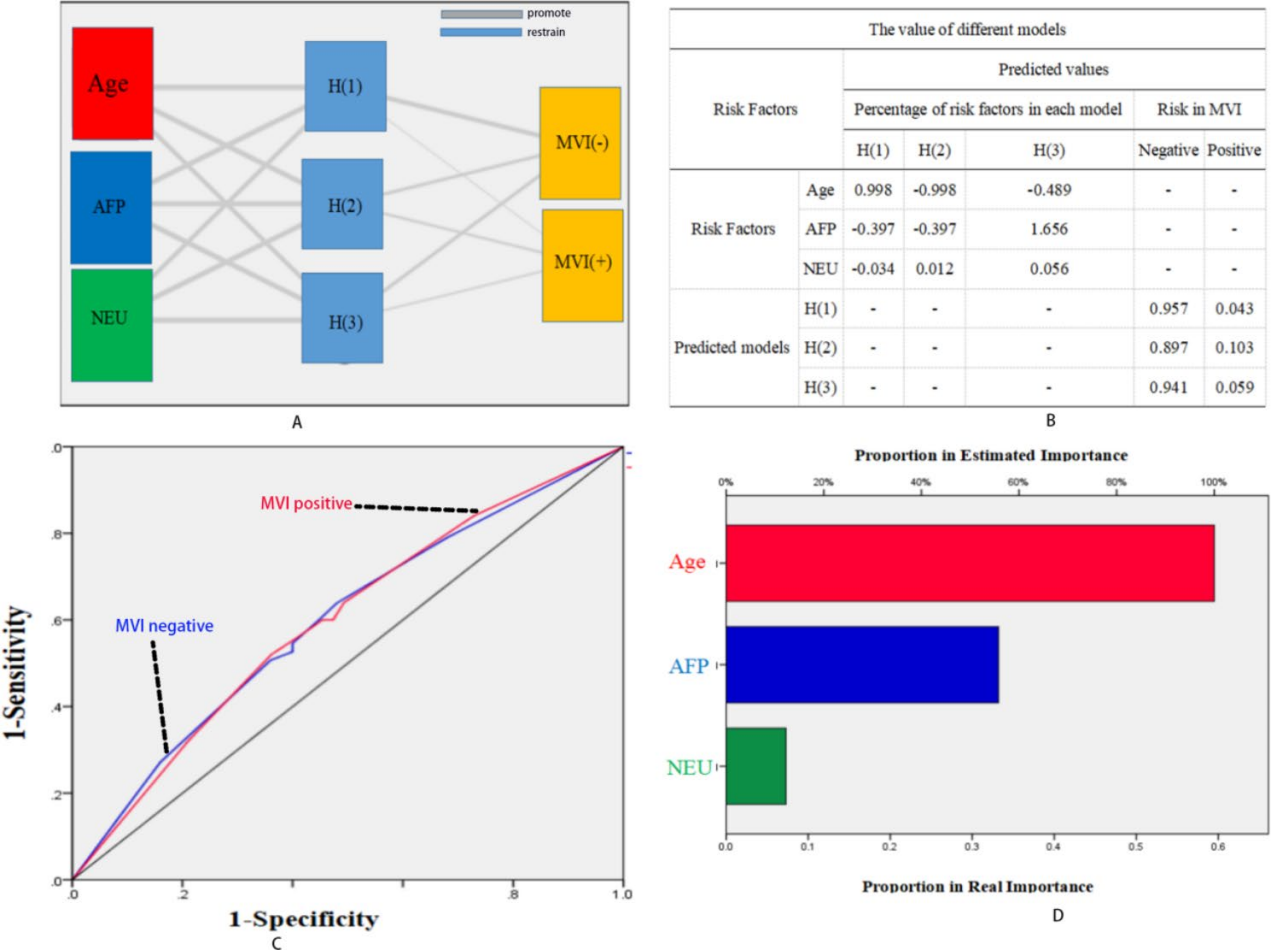
Neural network analysis for patients in the early stage with a single tumor

There were 382 patients with a single tumor in this study (diameter less than 3 cm). We performed univariable analysis, multivariable analysis and neural network analysis in these patients. We found that age and the levels of AFP and NEU were independent risk factors in these patients. Combined with Fig. 3-A, 3-B and 3-D, we found that the different risk factors had different effects on MVI status. Age was the most important risk factor for these patients, with an AUC = 0.605 (Fig. 3-C).

## Discussion

In the current study, we compared the differences between the MVI-positive group and MVI-negative group, and we found that age, multiple tumors, AFP level, NEU level and tumor diameter were independent risk factors for predicting MVI status. Age, as an independent risk factor for MVI status, might be associated with the long-term outcomes of elderly HCC patients [25]. In this study, the patients in the MVI-positive group were younger than the patients in the MVI-negative group, which was similar to the study carried out by Hao Xing et al. [25]. Thus, age could be a risk factor for predicting MVI status. However, its efficacy requires further research (Fig. 2-B and -D). On the other hand, MVI positivity might mean that few tumor cells have invaded into the blood, which would activate the immune system, resulting in an improved level of NEU. Tumor cells could not be eliminated completely; thus, there was a higher risk of tumor recurrence in the MVI-positive group [10–13], and the patient underwent hepatectomy. Therefore, the level of NEU was higher in the MVI-positive group. In this study, we divided the value of NEU (60%) according to Table 1, which was in the normal range. The incidence of MVI positivity reached 15-57.1% in a previous study [26], and the incidence of MVI was 13.84% in this study; thus, when we divided the level of NEU into a high level (> 70%), the AUC of the ROC was less than 0.5, which was limited by the small sample size. Therefore, the efficacy of NEU in predicting MVI status needs further research. The relationship was weak in predicting MVI positivity when the level of NEU was higher than 60%.

Previous studies [17, 18] reported that the number of nodules was an independent risk factor for tumor recurrence, which was mainly caused by metastatic recurrence from the main tumor via the portal system [19, 27]. Multiple tumors mean that the tumor would have invaded different sites of the portal vein system, especially for tumors located in different segments. Tumors located in different hepatic areas indicated that intrahepatic spread was more likely for tumors in different branches of the portal vein, resulting in higher recurrence and poor outcomes. On the other hand, multiple tumors were an independent risk factor in predicting MVI positivity, which may have a strong relationship between these two factors. MVI may be an early potential factor for intrahepatic metastasis, resulting in multiple nodules. Thus, it is meaningful for predicting MVI status following the number of tumors. Generally, the more quickly the tumor grows, the more aggressive it is. The diameter of the tumor might be a sign of tumor aggression, and taking the diameter into consideration for estimating the MVI status is reasonable.

A high level of AFP has been proven to have more aggressive behaviors in previous findings [28, 29], especially for levels of AFP higher than 1000 ng/mL. On the other hand, levels of AFP higher than 400 ng/mL were a risk factor for huge tumors [30, 31]. In the current study, the level of AFP was the most independent risk factor for MVI status (Fig. 2-D), which might be an explanation for the aggression of the high level of AFP. A previous study reported that AFP positivity was a predictor of poor prognosis in HCC after hepatic resection [32]. Therefore, an AFP level higher than 400 ng/ml might have a strong relationship with AFP positivity. It is a shortcoming that we did not perform genetic testing between these patients. However, the level of AFP could provide evidence for surgeons to estimate the MVI status before making a decision on the method of hepatectomy.

Certainly, it is difficult to predict the MVI status before hepatectomy. With the development of radiological techniques, surgeons prefer to perform RFA for early-stage HCC. Because the liver damage and the cost are less [33, 34], the patients would have a comparable outcome from RFA if we could identify the plenitudinous margin [35]. Thus, it is necessary to estimate the MVI status for early-stage HCC. In this study, we performed neural network analysis in patients with a maximum tumor diameter less than 3 cm and found that age was the most important risk factor for patients with a single tumor and a tumor diameter less than 3 cm (Fig. 3-D), with an AUC of 0.605 (Fig. 3-C). This means that when the patient is younger than 50 years old, the surgeon should take hepatectomy into first consideration. A previous study pointed out that hepatectomy for early-stage HCC has better long-term outcomes than RFA [36]. We have proposed that if we could identify the margin of RFA, we should also encourage these patients to receive RFA treatment [37]. On the other hand, previous studies did not divide patients with a tumor diameter less than 3 cm to analyze the risk factors for MVI positivity [17–19]. Thus, following the results of neural network analysis, we propose a hypothesis that young patients with a single tumor and a diameter less than 3 cm should undergo hepatectomy for a higher risk of MVI positivity.

The study also has other limitations. (1) This was a retrospective study and only a single-center experience with a small sample included, and the predicted models need further research to prove their application. (2) We did not compare the specificity and sensitivity with other prediction models. However, to the best of our knowledge, this study was the first to build predictive models to estimate MVI status by neural network analysis.

## Conclusion

The level of AFP is the most important risk factor for MVI-positive patients. Age is the most important risk factor for MVI positivity within a single tumor of early-stage HCC.

## Data Availability

The data sets used during the current study are available from the corresponding author on reasonable request.
